# Risk factors for HCV infection in Georgia: A case-control study

**DOI:** 10.3855/jidc.20658

**Published:** 2025-05-31

**Authors:** Maia Butsashvili, Giorgi Kanchelashvili, Ketevan Galdavadze, Maia Tsereteli, Davit Baliashvili, Senad Handanagic, Paige A Armstrong, Shaun Shadaker, George Kamkamidze

**Affiliations:** 1 Health Research Union, Tbilisi, Georgia; 2 Avicenna - Batumi Medical University, Batumi, Georgia; 3 National Center for Disease Control and Public Health, Tbilisi, Georgia; 4 The Task Force for Global Health, Tbilisi, Georgia; 5 Division of Viral Hepatitis, Centers for Disease Control and Prevention, Atlanta, GA, United States

**Keywords:** Hepatitis C, HCV transmission, injection drug use, invasive medical procedures, Georgia

## Abstract

**Introduction:**

Hepatitis C virus (HCV) infection is a leading cause of chronic liver disease, including cirrhosis and liver cancer. Prior studies in Georgia identified risk factors such as injection drug use (IDU), tattoos, dental cleanings, medical injections, and blood transfusion. This study explored risk factors associated with HCV seroconversion in Georgia.

**Methodology:**

A case-control study was conducted among adults aged ≥ 18 years. A total of 299 Cases (persons who seroconverted after ≥ 2 screenings) and 436 controls (persons with ≥ 2 negative anti-HCV test results dated 90 to 364 days apart) were randomly selected from the national HCV screening database from January 2019 to November 2020. Data were collected through telephone interviews and analyzed using descriptive statistics and logistic regression.

**Results:**

Among 206/299 (68.9%) cases and 229/436 (52.5%) controls who agreed to participate, 53.8% were female and 60.5% were aged > 40 years. After adjusting for covariates, independent predictors of HCV seroconversion were age > 40 years (adjusted odds ratio [aOR] = 2.47, 95% confidence interval [CI]: 1.52–4.01), male sex (aOR = 2.12, 95% CI: 1.34–3.34), IDU (aOR = 26.24, 95% CI: 3.27–210.43), history of invasive medical procedure (aOR = 3.19, 95% CI: 1.96–5.19), ≥ 24 hours of hospitalization (aOR = 2.01, 95% CI: 1.12–3.59), and occupational performance of any invasive medical procedure (aOR = 2.70, 95% CI: 1.12–6.53).

**Conclusions:**

Our findings suggest that HCV seroconversions in Georgia are associated with IDU, hospitalization, and invasive medical procedures. These identified risk factors provide opportunities to further improve the prevention of HCV infection in Georgia.

## Introduction

Hepatitis C virus (HCV) infection is a leading cause of chronic liver disease, including cirrhosis and liver cancer. In 2020, an estimated 56.8 million people were living with viremic HCV infection worldwide, with 641,000 patients initiating treatment [1]. Globally, HCV transmission is mostly associated with unsafe injections among persons who inject drugs (PWID), transfusion of unscreened blood, and invasive medical procedures, especially in developing countries. Sexual and mother-to-child transmission as well as sharing household sharp instruments can also contribute to HCV transmission [2]. Previous analysis showed that HCV exposure in Georgia was associated with injection drug use (IDU), tattoos, frequent dental cleanings, medical injections, multiple lifetime sexual partners, and blood transfusion [3–5].

In recent years, treatment of hepatitis C has undergone significant simplification, particularly with the availability of highly effective direct-acting antivirals (DAAs). These treatments are shorter, easier to administer, and achieve cure rates exceeding 95%, even in populations with advanced liver diseases such as cirrhosis. However, challenges remain in ensuring that all at-risk populations receive timely treatment, particularly marginalized groups like PWID and patients in rural areas. A key intervention includes the decentralization of care to increase accessibility of DAAs through community-based services and harm reduction programs. Moreover, while HCV elimination efforts have made progress, reinfection among PWID and other high-risk groups remains a challenge [6,7].

In 2015, Georgia launched the world’s first national hepatitis C elimination program. The Hepatitis C elimination program consists of prevention, diagnosis, and treatment of hepatitis C including improvements in infection prevention and control practices in health care and cosmetics settings and harm reduction services for PWID, among others. Up to March 2024, a total of 2.5 million adults were screened for antibodies to HCV (anti-HCV) and over 80,000 patients completed treatment and 99.0% were cured [8]. Since the implementation of the program, there has been a significant reduction in the prevalence of chronic HCV infection among the adult population in Georgia. A 2021 national serosurvey demonstrated a 67% reduction in prevalence of HCV infection, determined by nucleic acid test for detection of HCV RNA, among the adult population from 5.4% in 2015 to 1.8% in 2021 [3]. Understanding the primary sources of ongoing HCV transmission is crucial to focus prevention efforts and design targeted interventions. This study explored potential risk factors associated with HCV seroconversion in Georgia. It addresses gaps in knowledge of HCV transmission modes to inform targeted prevention efforts.

## Methodology

A case-control study was conducted among adults aged ≥ 18 years in 2022. Cases and controls were randomly selected from the national HCV screening database from January 1, 2019, to November 30, 2020. Cases were defined as persons who were tested for anti-HCV two or more times more than 12 days apart (to minimize misclassification from data entry errors) and seroconverted from anti-HCV non-reactive to anti-HCV reactive. Controls were defined as persons with ≥ 2 non-reactive anti-HCV test results dated 90 to 364 days apart with no subsequent reactive test. From January 1, 2019, to November 30, 2020, a total of 1,008 seroconversion cases were identified. Out of them, 803 had phone numbers available through COVID-19 and HCV elimination databases. The sample size calculation was performed using the methodology for descriptive studies for the expected proportion of seroconversion of 0.50, degree of accuracy (margin of error) +/− 0.05, 95% confidence level, and the corresponding population size (803). A non-response rate of 15% was also considered for the calculation of the total number of study subjects for inclusion in the study. Consequently, the sample size was 299 cases. Out of the 299 selected cases, 5 had died and 80 had incorrect telephone numbers, resulting in 214 successfully contacted individuals. Among them, 8 refused to participate. We aimed for a 1:2 ratio of cases and controls. The sampling frame for controls included 241,877 adults, from which 436 controls were randomly selected. Both cases and controls provided verbal consent and were then asked to participate in a telephone interview. Data on participants who refused to participate in the study were not collected. The interviews were conducted by trained epidemiologists from Georgia’s National Centre for Disease Control and Public Health (NCDC).

The questionnaire collected socio-demographic, clinical, and behavioral information (see supplementary material 1 for details). Cases were surveyed in 2020 and were asked about exposure to risk factors for HCV infection in the 6 months preceding their reactive HCV antibody test. Controls were surveyed in 2022 and data were collected for 2–6 months preceding the interview. For the controls, individuals who had a reactive anti-HCV test at any time before the selection process in 2022 were excluded.

Descriptive statistics were used to characterize study participants. Odds ratios (OR) and 95% confidence intervals (CI) were calculated to evaluate the association between HCV seroconversion status and different socio-demographic, clinical, and behavioral variables. Binary logistic regression analysis was performed to identify independent predictors of HCV seroconversion. Covariates that were significant in bivariate analysis were entered into the multivariable model and fit was evaluated by Hosmer-Lemeshow test to produce adjusted odds ratios (aOR). To assess potential collinearity among variables such as history of blood transfusion, ≥ 24 hours hospitalization, and history of any surgery or invasive medical procedure in the past 12 months, we performed Spearman correlation analysis to estimate the association between all three variables. The correlation coefficients for all comparisons were lower than 0.3, indicating low potential collinearity among these variables. Therefore, these variables were retained in the multivariable model. Denominators for behavioral characteristics include only those who gave a definitive answer to each question. Data entry, processing, and statistical analysis were performed using the SPSS v.23.0 statistical package (Chicago, IL).

## Results

A total of 206 cases and 229 controls participated in the survey; response rates were 68.9% (n = 206/299) and 52.5% (n = 229/436), respectively. Overall, 53.8% (n = 234) of participants were female and 60.5% (n = 263) were aged > 40 years. Participants’ median age (interquartile range) was 46 (35–61). During the months surveyed, 37.2% (n = 154/414) had surgery or an invasive medical procedure such as dental surgery, endoscopy, gynecological, or other, 20.7% (n = 86/415) spent ≥ 24 hours in the hospital, 4.8% (n = 20/413) injected drugs, 3.9% (n = 16/415) received a blood transfusion, and 7.1% (n = 30/422) performed an invasive medical procedure as a part of their occupation. Fifteen respondents (3.5%, n = 15/425) reported that they had been vaccinated against hepatitis B (Table 1).

### Comparison of cases and controls

In bivariate analysis, cases were more likely than controls to be male (57.8% vs. 35.8%; OR = 2.45, 95% CI: 1.67–3.61) and aged > 40 years (69.4% vs. 52.4%; OR = 2.06, 95% CI: 1.39–3.06) (Table 1). Receipt of a blood transfusion was more prevalent among cases (7.0% vs. 1.3%; OR = 5.66, 95% CI: 1.59–20.18), as was IDU (10.3% vs. 0.4%; OR = 26.25, 95% CI: 3.48–198.10). Cases were more likely to have undergone surgery or an invasive medical procedure (50.8% vs. 26.2%; OR = 2.91, 95% CI: 1.93–4.39) and to have spent ≥ 24 hours in the hospital (31.7% vs. 11.8%; OR = 3.48, 95% CI: 2.09–5.77) during the relevant survey period. Cases were more likely to have had any invasive medical procedure as a part of their occupation (9.7% vs 4.6%; OR = 2.22, 95% CI: 1.01–4.85). Seven (3.8%) cases were incarcerated or detained compared to no controls.

In multivariable analysis, after adjusting for sex, age, IDU, surgery or an invasive medical procedure, spending ≥ 24 hours in the hospital, any invasive medical procedure as a part of their occupation, and receipt of a blood transfusion, the independent predictors of HCV seroconversion were age > 40 years (aOR = 2.47, 95% CI: 1.52–4.01), male sex (aOR = 2.12, 95% CI: 1.34–3.34), IDU (aOR = 26.24, 95% CI: 3.27–210.43), surgery or an invasive medical procedure (aOR = 3.19, 95% CI: 1.96–5.19), ≥ 24 hours of hospitalization (aOR = 2.01, 95% CI: 1.12–3.59), and performing any invasive medical procedure as a part of their occupation (aOR = 2.70, 95% CI: 1.12–6.53) (Table 1).

## Discussion

The study found that males and individuals over 40 years old were more likely to have HCV seroconversion, indicating recent HCV infection. This is consistent with a previous study that has shown that older age and male sex are associated with HCV infection [9]. The recent nationwide hepatitis C serosurvey conducted in 2021 in Georgia also reported that the prevalence of HCV infection was higher among men than women and the highest rates of infection (HCV RNA positive) were observed in individuals aged 40–49 years [3].

This study also found that having or performing surgery or an invasive medical procedure and hospitalization for ≥ 24 hours was associated with HCV seroconversion. A previous study in Georgia has also reported similar findings [5]. The risk of HCV transmission through these routes can be minimized by implementing proper infection prevention and control (IPC) practices in healthcare settings. The Ministry of Labor, Health, and Social Affairs of Georgia aims to strengthen the IPC program. A 2018 assessment revealed gaps in IPC programs in hospitals, including inadequate staff training, limited monitoring, and missing comprehensive IPC guidelines. Challenges included a lack of standardized protocols, training on hand hygiene, regular disinfection and sterilization of medical equipment, and adherence to standard precautions during medical procedures [10].

The finding that injection drug use was a significant risk factor for HCV seroconversion is consistent with previous research and is a major concern globally [11]. Georgia’s HCV elimination program has prioritized harm reduction services, such as needle/syringe services programs and opioid substitution therapy. Decentralization of HCV treatment has also been implemented, which has shown positive outcomes in reducing exposure to and prevalence of HCV infection among PWID [3]. The 2021 HCV serosurvey found a significant decline in chronic HCV infection prevalence among persons who ever injected drugs, from 51.1% in 2015 to 17.8% in 2021 [3]. The high prevalence of injection drug use among HCV seroconversion cases highlights the continued need for education on safe injection practices and substance use disorder treatment to prevent HCV transmission among PWID.

The identified risk factors, such as injection drug use and hospitalization, align with Georgia’s HCV elimination strategies, particularly in the emphasis on harm reduction and infection prevention. However, our findings highlight the need to expand prevention services for high-risk groups like PWID, and further strengthen IPC protocols in healthcare settings to reduce HCV transmission.

Our findings suggest that while the HCV elimination program has made significant progress, targeted interventions for high-risk populations are necessary to further reduce transmission and achieve the goals of the HCV elimination. The experience of Georgia serves as a model for other countries pursuing HCV elimination, particularly in terms of integrating harm reduction and healthcare system improvements.

The study had limitations. The response rate among control subjects was low. The risk profile of those who refused to participate in the control group is unclear due to the lack of data collected for these individuals. Their non-participation may introduce selection bias, as they may differ from those who agreed to participate with respect to risk factors. The study population was limited to adults aged 18 years and older, which may not be representative of the entire population at risk of HCV infection. The small number of participants reporting some risk behaviors (e.g., IDU and blood transfusion) led to wide confidence intervals so results should be interpreted with caution. Additionally, the study questionnaire did not ask about detailed history of specific medical interventions, limiting our ability to identify specific types of surgeries and invasive medical procedures that might be associated with the risk of HCV infection. Furthermore, for controls, the questionnaire asked about risk factors 2–6 months preceding the interview and for cases, exposure information was collected during the last 6 months before the reactive antibody test result, but the actual timing of infection is unknown, which could result in exposures outside that time interval. Lastly, our findings may be biased due to recall and/or social desirability biases.

## Conclusions

In conclusion, our findings suggest that HCV seroconversions in Georgia are associated with injection drug use, hospitalization and invasive medical procedures. Improving IPC practices in health care settings and increasing coverage of harm reduction services for PWID are key interventions needed to reduce HCV transmission in Georgia. These results provide opportunities to target known transmission sources for HCV seroconversion and further prevent HCV infection in Georgia. Future research should focus on understanding reinfection patterns among PWID, evaluating the long-term effectiveness of prevention and treatment programs, and addressing healthcare-associated transmission to refine intervention strategies.

## Supplementary Material

1

## Figures and Tables

**Table 1.** Socio-demographic characteristics of cases and controls and factors associated with HCV seroconversion, Georgia, 2021.

Characteristics	Total N = 435, n (column %)	Cases N = 206, n (column %)	Controls N = 229, n (column %)	OR (95% CI)	Adjusted* OR (95% CI)
**Sex**					
Male	201 (46.2%)	119 (57.8%)	82 (35.8%)	2.45 (1.67–3.61)	2.12 (1.34–3.34)
Female	234 (53.8%)	87 (42.2%)	147 (64.2%)		
**Age**					
≤ 40 years	172 (39.5%)	63 (30.6%)	109 (47.6%)	2.06 (1.39–3.06)	2.47 (1.52–4.01)
> 40 years	263 (60.5%)	143 (69.4%)	120 (52.4%)		
As a part of their occupation, they performed any invasive medical procedure**	30/422 (7.1%)	20/206 (9.7%)	10/216 (4.6%)	2.22 (1.01–4.85)	2.70 (1.12–6.53)
Vaccinated against hepatitis B**	15/425 (3.5%)	6/201 (3.0%)	9/224 (4.0%)	0.74 (0.26–2.10)	-
Injection drug use**	20/413 (4.8%)	19/184 (10.3%)	1/229 (0.4%)	26.25 (3.48–198.10)	26.24 (3.27–210.43)
Received a blood transfusion**	16/415 (3.9%)	13/186 (7.0%)	3/229 (1.3%)	5.66 (1.59–20.18)	3.35 (0.79–14.18)
Spent ≥ 24 hours in the hospital**	86/415 (20.7%)	59/186 (31.7%)	27/229 (11.8%)	3.48 (2.09–5.77)	2.01 (1.12–3.59)
Had any type of surgery or invasive medical procedure (dental surgery, endoscopy, gynecological)**	154/414 (37.2%)	94/185 (50.8%)	60/229 (26.2%)	2.91 (1.93–4.39)	3.19 (1.96–5.19)
Got a tattoo or piercing**	11/413 (2.7%)	5/186 (2.7%)	6/227 (2.6%)	1.02 (0.31–3.39)	-
Got a manicure or a pedicure at beauty salons**	52/406 (12.8%)	18/184 (9.8%)	34/222 (15.3%)	0.60 (0.33–1.10)	-
Incarcerated or detained in prison or jail**	7/407 (1.7%)	7/185 (3.8%)	0/222 (0.0%)	-	-
Treated for sexually transmitted disease(s) (Syphilis, gonorrhea, chlamydia, trichomoniasis)**	7/409 (1.7%)	4/185 (2.2%)	3/224 (1.3%)	1.63 (0.36–7.37)	-
Had unprotected sex (without condom) with HCV infected partner**	10/378 (2.6%)	6/156* (3.8%)	4/222* (1.8%)	2.18 (0.60–8.86)	-

OR: odds ratio; CI: confidence interval.

* Adjusted for sex, age, injection drug use, surgery or an invasive medical procedure, spending ≥ 24 hours in the hospital, any invasive medical procedure as a part of their occupation, blood transfusion.

** Numbers and percentages include participants who answered yes (numerator) and those who answered yes or no (denominator); participants who refused to answer or answered “Don’t know” were excluded from calculations so some denominators do not add up to total N. For each characteristic, reference group was control group.

## References

[1] Polaris observatory HCV collaborators (2022) Global change in hepatitis C virus prevalence and cascade of care between 2015 and 2020: a modelling study. Lancet Gastroenterol Hepatol 7: 396–415. doi: 10.1016/S2468-1253(21)00472-6. 35180382

[2] WHO (2022) Global health sector strategies on HIV, viral hepatitis and the sexually transmitted infections. Available: https://www.who.int/publications/i/item/9789240053779#:~:text=Overview-,Global%20health%20sector%20strategies%20on%2C%20respectively%2C%20HIV%2C%20viral%20hepatitis,sexually%20transmitted%20infections%20by%202030. Accessed: July 18, 2022.

[3] Gamkrelidze A, Shadaker S, Tsereteli M, Alkhazashvili M, Chitadze N, Tskhomelidze I, Gvinjilia L, Khetsuriani N, Handanagic S, Averhoff F, Cloherty G, Chakhunashvili G, Drobeniuc J, Imnadze P, Zakhashvili K, Armstrong PA (2021) Nationwide hepatitis C serosurvey and progress towards hepatitis C virus elimination in the country of Georgia. J Infect Dis 228: 684–693. doi: 10.1093/infdis/jiad064. PMC10506179 36932731

[4] Hagan LM, Kasradze A, Salyer SJ, Gamkrelidze A, Alkhazashvili M, Chanturia G, Chitadze N, Sukhiashvili R, Shakhnazarova M, Russell S, Blanton C, Kuchukhidze G, Baliashvili D, Hariri S, Ko S, Imnadze P, Drobeniuc J, Morgan J, Averhoff F (2019) Hepatitis C prevalence and risk factors in Georgia, 2015: setting a baseline for elimination. BMC Public Health 19: 480. doi: 10.1186/s12889-019-6784-3. 32326913 PMC6696670

[5] Baliashvili D, Averhoff F, Kasradze A, Salyer SJ, Kuchukhidze G, Gamkrelidze A, Imnadze P, Alkhazashvili M, Chanturia G, Chitadze N, Sukhiashvili R, Blanton C, Drobeniuc J, Morgan J, Hagan LM (2022) Risk factors and genotype distribution of hepatitis C virus in Georgia: A nationwide population-based survey. PLoS One 17: e0262935. doi: 10.1371/journal.pone.0262935. 35061841 PMC8782338

[6] Abu-Freha N, Mathew Jacob B, Elhoashla A, Afawi Z, Abu-Hammad T, Elsana F, Paz S, Etzion O (2022) Chronic hepatitis C: Diagnosis and treatment made easy. Eur J Gen Pract 1: 102–108. doi: 10.1080/13814788.2022.2056161. PMC9116263 35579223

[7] Taha G, Ezra L, Abu-Freha N (2023) Hepatitis C elimination: opportunities and challenges in 2023. Viruses 15: 1413. doi: 10.3390/v15071413. 37515101 PMC10386528

[8] CDC, Viral hepatitis divison (2024) 9th viral hepatitis technical advisory group meeting, Georgia hepatitis C elimination program care cascade. Available: https://www.cdc.gov/hepatitis/global/georgia-elimination-program.html. Accessed: September 3, 2024.

[9] Mohd Hanafiah K, Groeger J, Flaxman AD, Wiersma ST (2013) Global epidemiology of hepatitis C virus infection: new estimates of age-specific antibody to HCV seroprevalence. Hepatology 57: 1333–42. doi: 10.1002/hep.26141. 23172780

[10] Deryabina A, Lyman M, Yee D, Gelieshvilli M, Sanodze L, Madzgarashvili L, Weiss J, Kilpatrick C, Rabkin M, Skaggs B, Kolwaite A (2021) Core components of infection prevention and control programs at the facility level in Georgia: key challenges and opportunities. Antimicrob Resist Infect Control 10: 39. doi: 10.1186/s13756-020-00879-3. 33627194 PMC7903395

[11] Nelson PK, Mathers BM, Cowie B, Hagan H, Des Jarlais D, Horyniak D, Degenhardt L (2011) Global epidemiology of hepatitis B and hepatitis C in people who inject drugs: results of systematic reviews. Lancet 378: 571–83. doi: 10.1016/S0140-6736(11)61097-0. 21802134 PMC3285467

