# Supplementary material for: Risk factors for HCV infection in Georgia: A case-control study
Source: J Infect Dev Ctries. Author manuscript; Available in PMC 2026 May 31. (PMC12146773; doi:10.3855/jidc.20658)
Supplement: 1 [file NIHMS2087035-supplement-1.pdf]

Annex – Supplementary Items

Supplementary material 1. Behavioral questionnaire for cases and controls.

**Study of risk factors for hepatitis C**		
Completion date:		
Participant ID:		
Interviewer:		
**No.**	**Question**	**Answer**
1.	Age	
2.	Sex	1. Male<br>2. Female 3.
3.	Address	Region ------------------------<br>City/Town --------------------------
4.	Level of education	1. Incomplete secondary<br>2. Complete secondary<br>3. Vocational<br>4. Incomplete higher education<br>5. Complete higher education<br>99. Did not answer
5.	Nationality	1. Georgian<br>2. Armenian<br>3. Azerbaijani<br>4. Russian<br>5. Assyrian<br>6. Other________________<br>99. Did not answer
6.	What was your occupation during last 3 years? Choose all that apply.<br>**(It is possible to select several options)**	1. Healthcare<br>Specify------------------------<br>2. Education<br>3. Tourism<br>Specify-------------------------<br>4. Transportation and delivery<br>Specify-------------------------<br>5. Police<br>6. Postal worker, delivery service<br>7. Airports<br>Specify-----------------<br>8. Agriculture<br>9. Accommodation and food service<br>Specify-----------------<br>10. Manufacturing and construction<br>11. Information communication technology<br>12. Salespersons<br>13. Others<br>Specify-----------------<br>99. Did not answer
7.	As a part of your occupation, did you perform any invasive medical procedure (exposure prone procedure, assisting a surgery, administrating intravenous or intramuscular injections or in invasive dental procedure.)	1. Yes<br>2. No<br>99 Didn't answer
8.	Have you been vaccinated against hepatitis B?	1. Yes (If yes please indicate the year----------)<br>2. No<br>99. Didn't answer
**For cases - following questions address general and potential risk factors for the transmission of hepatitis C. Therefore, please recall the period of 2 - 6 months before being diagnosed with Hepatitis C.**<br>**For controls - following questions address general and potential risk factors for the transmission of hepatitis C. Therefore, please recall the period of last 2 - 6 months of your life.**		
9.	Did you undergo hemodialysis?	1. Yes<br>2. No<br>99. Didn't answer
10.	Did you inject drugs?	1. Yes<br>2. No<br>99. Didn't answer
11.	Did you receive a blood transfusion?	1. Yes<br>2. No

		99. Didn't answer
12.	Did you spend **≥ 24 hours** or more in the hospital?	1. Yes<br>2. No<br>99. Didn't answer
13.	Did you have any type of surgery or invasive medical procedure (dental surgery, endoscopy, gynecological)?	1. Yes<br>2. No<br>99. Didn't answer
14.	Did you get a tattoo or piercing during the above-mentioned period?	1. Yes<br>2. No<br>99. Didn't answer
15.	Did you get a manicure or a pedicure at beauty salons?	1. Yes<br>2. No<br>99. Didn't answer
16.	When you go to the barber do you know whether you are being shaved with new razors, or razors that have been used before?<br>**(Men Only)**	1. New<br>2. Used<br>3. Used and sterilized<br>4. Razor was cleaned in a solution<br>5. Was in a package after being sterilized<br>6. Doesn't get shaved at the barber.<br>7. Doesn't remember
17.	Were you incarcerated or detained in prison or jail?	1. Yes<br>2. No<br>99. Didn't answer
18.	Were you treated for sexually transmitted disease(s)? (Syphilis, gonorrhea, chlamydia, trichomoniasis)	1. Yes<br>2. No<br>99. Didn't answer
19.	Have you had unprotected sex **(without a condom)** with HCV infected partner?	1. Yes<br>2. No<br>99. Didn't answer
